# Brazilian union actions for workers’ health protection

**DOI:** 10.1590/S1516-31802005000100006

**Published:** 2005-01-02

**Authors:** Rodolpho Repullo, Jorge da Rocha Gomes

**Keywords:** Occupational health, Social medicine, Labor unions, Regulations, Saúde ocupacional, Medicina social, Sindicatos de trabalhadores, Regulamentos

## Abstract

**CONTEXT:**

Many authors have emphasized the importance of worker strength through unionized organizations, in relation to the improvement of working procedures, and have reported on the decisiveness of labor movement actions in achieving modifications within the field of work and health.

**OBJECTIVE:**

To describe the ways in which Brazilian unions have tried to intervene in health-illness and work processes, identifying the existence of commonality in union actions in this field.

**TYPE OF STUDY:**

Qualitative study.

**SETTING:**

Postgraduate Program, Environmental Health Department, Faculdade de Saúde Pública, Universidade de São Paulo, São Paulo, Brazil.

**METHODS:**

Union health advisers and directors were interviewed. Documents relating to union action towards protecting workers’ health were collected and analyzed.

**RESULTS:**

Unions articulate actions regarding workers’ health of a technical and political nature that involve many aspects and high complexity. These have been divided into thematic categories for better analysis.

**DISCUSSION:**

Union actions regarding workers’ health in Brazil are restricted to some unions, located mainly in the southern, southeastern and northeastern regions of the country. Nonetheless, the unions undertaking such actions represent many professions of great economic and political importance.

**CONCLUSIONS:**

The recent changes in health and safety at work regulations, recognition of professional diseases, creation of workers’ health services and programs within the unified health system, and operational improvements in companies’ specialized safety and occupational medicine services, all basically result from union action. There is commonality of union action in this field in its seeking of technical and political strengthening for all workers and their general and local representation. This has the objective of benefiting collective bargaining between employers and workers. Inter-institutional action on behalf of workers’ rights guarantees and amplifies the improvement of health and working conditions.

## RESUMO

**CONTEXTO:**

Vários autores enfatizam a importância do fortalecimento do trabalhador através das organizações sindicais, na melhoria dos processos de trabalho e relatam a ação decisiva do movimento sindical nas transformações no campo da saúde e trabalho.

**OBJETIVO:**

Descrever as formas que os sindicatos brasileiros utilizam para intervir no processo saúde-doença e trabalho, identificando a existência de uma modalidade comum de atuação sindical na área.

**LOCAL:**

Programa de Pós-Graduação. Departamento de Saúde Ambiental. Faculdade de Saúde Pública da Universidade de São Paulo – São Paulo – Brasil.

**MÉTODO:**

Foram realizadas entrevistas com técnicos de saúde dos sindicatos e diretores sindicais. Foram coletados documentos relacionados com a ação sindical na proteção da Saúde do Trabalhador e o material analisado.

**RESULTADOS:**

Na ação sindical em Saúde do Trabalhador existem ações articuladas de natureza técnica e política que envolvem muitos aspectos e um alto grau de complexidade. Para que a análise seja melhor, esses diferentes aspectos da ação sindical são divididos nas categorias temáticas utilizadas para classificar o material coletado.

**DISCUSSÃO:**

As ações sindicais voltadas para a Saúde do Trabalhador no Brasil estão restritas a alguns sindicatos, localizados principalmente nas regiões Sul, Sudeste e Nordeste do País. Apesar disso, os sindicatos que desenvolvem essas ações, são os que representam categorias numerosas, de grande importância econômica e política.

**CONCLUSÕES:**

As recentes mudanças na legislação de Saúde e Segurança no Trabalho, o reconhecimento das doenças profissionais, a criação de programas e serviços de Saúde do Trabalhador no Sistema Único de Saúde e a melhoria da ação profissional nos Serviços Especializados em Segurança e Medicina do Trabalho nas empresas, basicamente resultam dessa atuação. Existe uma modalidade comum de ação sindical na área e esta possui um componente que é a busca do fortalecimento técnico e político dos trabalhadores e da sua representação geral e local. Ela tem um objetivo que é beneficiar os acordos coletivos entre patrões e empregados e a ação interinstitucional em prol da garantia e ampliação dos direitos dos trabalhadores na melhoria das condições de saúde e trabalho.

## INTRODUCTION

There has been Brazilian working class involvement in health issues, especially those related to working conditions, since the beginning of the twentieth century. But it was only in the late 1970s that trade unions began to act more significantly within this field. These trade union actions have developed into large initiatives, both in technical and in political fields, to the point that Bedrikow^1^ has considered such actions as the only achievement within Brazilian occupational medicine over the last few years.

Many authors have emphasized the importance of worker strength through unionized organizations, in relation to the improvement of working procedures. Several authors have reported on the decisiveness of labor movement actions in achieving modifications within the field of work and health, either related to public compensation and labor legislation or to the direct relationship between employers and employees.^2-11^

In Latin America, the trade union struggle for better working conditions and health is of recent origin and is still influenced by the lack of guarantees at work, by excessively paternalistic assistance and lack of democracy in the unions. It is also influenced by the priority given to economic issues, to the detriment of all issues regarding health and working conditions. Such influences result from pressure by employers and the state, and from other situational-structural factors within the economies of peripheral capitalist countries.^12-14^

It is important to emphasize that in Brazil there are two types of trade unions: unions for categories of workers and professional associations.^15^ The relative delay among unions in entering into the fight for better working conditions and health is attributed by Mendes^16^ to impedance either through direct intervention of the State or through cooptation, thereby causing unions to keep their actions turned towards paternalistic assistance activities, especially with regard to health issues.

The unleashing of actions involving workers’ health and safety matters by the Brazilian labor movement that began in the 1980s is attributed by Dias^17-18^ to the emergence of new unionism, grounded in the expansion of basic contacts through working on daily issues in workers’ lives, and to the appearance of new union practices regarding health that translated into claims for better working conditions along the guidelines of collective bargaining. Among these new union practices on health was the increasing union influence on the workplace through "health weeks", specific meetings and debates about the issue. Together with this was union participation in the process of modification of the legislation, as happened in the Eighth National Health Conference, the National Constitutional Assembly and the discussion of the organizational law regarding health.

The Inter-union Study and Research Department for the working environment and health was founded on June 14, 1981, by a decision of workers’ federations and trade unions in the State of São Paulo, with the purpose of assisting the labor movement in its studies and actions regarding working environments and conditions. This facility has improved the quality of the workers’ health movement by means of providing unions with the option of being able to call upon specialized technical assistance to guide their struggles.^19^ Such technical support has also been made possible by unions’ direct hiring of specialized professionals who are committed towards workers’ health and responsible for the preparation of studies and investigations and the dissemination of accumulated knowledge. This is a continuous process for socializing information and restoring and systematizing "workers’ knowledge".^17^

From a legal point of view, workers’ participation in occupational health and safety matters is assured by Law No. 6514/77, as regulated by Decree No. 3214/78 from the Ministry of Labor. This determines the need to implement accident prevention committees within private and public enterprises, and within governmental bodies whose employees are governed by the consolidated labor laws. Such internal accident prevention committees are made up partly by employer representatives and partly by members elected by employees. The size of the committee is determined according to the number of employees and the degree of risk within the enterprise. These committees are chaired by one of the employer’s representatives.

The Italian workers’ model, as translated from the book by Oddone et al.,^20^ has decisively influenced union actions in Brazil and Latin America. This methodology has led to many achievements in union intervention.^21-24^ The creation of the National Institute for Health at Work, by means of a convention between the Brazilian National Workers’ Federation (Central Única dos Trabalhadores) and the Italian General Confederation of Workers, in December 1990, has strengthened this influence. Also, unions not tied to Central Única dos Trabalhadores have drawn great influence from the Italian workers’ model in their actions.

The objective of this work was to describe the ways in which Brazilian unions attempt to intervene in health-illness and work processes, with the identification of common factors in the methods utilized in union action within these fields.

## METHODS

The exploratory phase of this work consisted of interviews with unions’ health advisers and union directors, and collecting documents from journalistic, union and academic sources, in relation to union action for protecting workers’ health.

Open, unstructured interviews were held with the purpose of collecting the official line on action on health and work from union directors. Nine directors and six advisers responsible for health issues were interviewed. These interviewees represented eight unions and two health and work union advisory bodies. The major themes included in the interviews concerned the methods used for informing, mobilizing and educating members of their categories of workers, and for strengthening local representation, collective bargaining and action within inter-institutional forums, and the structures the unions have for such action. These themes were chosen because they constitute the theoretical model for union action formulated by the present authors, based on their own experiences. One of the authors was formerly an adviser to one of the more important unions in the country.

These unions were chosen by the fact that they have recognizably acted in protecting workers’ health and have been cited in publications because of such.^25-29^ They also represent different concepts and practices from a socioeconomic and political point of view within the Brazilian labor movement at a national level, in relation to important categories of workers. Six of the unions are affiliated to the major Brazilian central union body, Central Única dos Trabalhadores, and belong to their different political factions. Another is affiliated to "Union Strength" (Força Sindical) and the remainder are affiliated to the General Workers’ Federation (Central Geral dos Trabalhadores). Four of them are from the Metropolitan Region of São Paulo, which is the most industrialized of the country. The others are from Rio de Janeiro, Minas Gerais, Rio Grande do Sul and Bahia, which are, with São Paulo, the most industrialized states in Brazil.

Material related to union action on workers’ health was collected from non-governmental organizations that are dedicated to study of the Brazilian labor movement, and from the authors’ personal files. Nine other organizations were thus added to the list of unions under examination. Seven are affiliated to Central Única dos Trabalhadores, one to Força Sindical and the other is not affiliated to central union bodies. Seven are from the state of São Paulo, one from Rio de Janeiro and the other from Minas Gerais. Eleven of all the unions studied represent categories of workers within the secondary sector of economy (metal, chemical, textile, building and food workers) and the others are from the service sector, including one representing public sector workers.

The analysis of the material collected was undertaken using the method put forward by Minayo,^30^ with observation of the following steps:

1. *Data systematization*: which included the interview material as well as the documents collected and the observation material.
2. *Data classification*: the repeated reading of texts to ascertain the main ideas relevant to the issue yielded thematic categories that were established on the basis these themes were plentifully cited or referred to in the material collected and represented different aspects of union action in the protection of workers’ health. Thus, the information was classified into the following themes:
  - Medical care;
  - Development and communication of critical awareness;
  - Mobilization;
  - Education;
  - Strengthening of the organization in the workplace;
  - Collective bargaining;
  - Inter-institutional action on workers’ health;
  - Structures for action on workers’ health;
  - Influences on actions.
3. *Model elaboration and final analysis*: the facts revealed through the results from the earlier procedures were laid out in a description of Brazilian union intervention methods on health and work issues.

## RESULTS AND DISCUSSION

In Brazil, union action on workers’ health is restricted to very few unions, as can be seen from an interview with a director of Central Única dos Trabalhadores:

*"We have 36 unions acting on workers’ health, represented in the National Health Collective. Within Central Única dos Trabalhadores, these are the most active unions out of the 2,300 branch unions. Today, in the Collective, we have 17 states that are organized with State Health Collectives, and which have representatives on the National Collective."*

Within union action on workers’ health, actions of a technical and political nature are articulated that involve many matters and have a high degree of complexity. For better analysis, these different aspects of union action will be presented in the thematic categories utilized to classify the material collected.

### 1. Medical care

Some concern was identified about the traditions of paternalistic assistance among Brazilian unions regarding health issues, as in the following interview passage:

*"After the days of the military dictatorship, with the repression, the union was obliged — and we should say that this was the history of all Brazilian unions, especially ours — to provide medical and dental care. In fact, I believe it consumed about 70% or almost 80% of the union’s whole resources."*

Healthcare extended to the worker and his family, as an obligation demanded by the Brazilian union organization, is being progressively replaced by actions on workers’ health. A physician working for one of the unions studied said that, after healthcare had been identified as a problem to be solved in the union and there came to be a desire for withdrawing it from the union’s practices, what was done was not the simple winding up of all attendance but the replacement of general medical care by special attention to workers’ health. Attending doctors whose activities were directed towards associates and their dependents were replaced by doctors committed to workers’ health.

In another union, the solution found for solving the problem of the cost of medical attendance was to create a Foundation that would manage medical insurance for its associates. In yet another union, which had two medical departments, one for workers’ health action and the other for medical care, this latter was simply wound up, while the former was maintained.

All the unions studied are concerned about the attendance that is being provided to their associates. This medical and dental activity is part of the unions’ legal structure from the 1940s that has been progressively disrupted by changes in the law and union practices. Paternalistic assistance on health issues is being substituted by union practices on workers’ health.

#### 3.2. Development and communication of critical awareness

All the union entities studied showed their concern for information and the development of critical consciousness regarding health-related matters and protection of workers on duty, either through an interview or in the material collected, as is described in the extract below, from an interview with a director of one of the unions:

*"Communication with this professional category is made by bulletins. For example, a problem is detected and so we complain about it. We write a letter, we demand that the company solve it. Our policy here is really to complain! Starting from the moment we issue a warning, they move."*

The bulletin is the main communication instrument with the rank and file of the professional category, and this is very effective. The utilization of bulletins, apart from providing information, provides motivation for debate. By making the problem collective and raising awareness of its effect upon workers’ health, it can be expected that management will be forced to solve the problem because of the public outcry.

The following is part of an interview with the director of the Osasco Metal Workers’ Union,^27^ who also deals with the information issue by distributing a report to workers:

*"Among all our daily tasks, there is the delivery of a weekly report that every metal worker receives. It doesn’t matter if it’s a three-thousand-employee company or a five-employee company."*

Conversations with workers, in a regular and planned manner, in previously programmed situations such as the day of medical consultations, and the utilization of daily or weekly radio programs, are other ways the unions use for communicating with their professional categories, as was seen in some of the interviews. The subjects of the information material produced by unions that were mentioned were very diverse. They included information about certain risks, such as exposure to lead, benzene, asbestos, occupational accidents and noise, and provided information regarding the law and workers’ rights, in relation to enterprises and government institutions. Analysis of the material collected that was utilized by unions in its information about workers’ health showed that the news items were presented in a format that was suitable for reading during work breaks or while commuting to and from work.

#### 3.3. Mobilization

Campaigns are a very normal way of mobilizing the professional category regarding issues related to workers’ health, according to the data from the collected material. In Brazil, unions’ most important events are salary campaigns. In terms of the organization, material and human resources involved, such campaigns are only comparable with union elections, especially those involving large disputes. In one of the articles collected from union publications,^31^ union leaders try to generate the same mobilization as in salary campaigns, but this time for better health and working conditions.

Union campaigns are planned in the same way as major advertising operations, with the creation of buzzwords and slogans first of all. Following this, videos, stickers, manuals, pamphlets, posters and other printed materials are produced. Then, seminars are scheduled, along with conferences and mass activities. The objective of these campaigns, aside from the problems complained about, is the modification and elimination of harmful working environments. Such campaigns always search for support and joint action with social sectors and institutions, whether governmental or not, who can ally themselves to the process, so as to strengthen it. The activities performed and advertising material produced are frequently addressed to these sectors.

In the material collected, many other campaigns were identified concerning the relationship between health and work. Apart from campaigns, unions organize permanent health committees. These have periodic meetings involving not only union directors and technical experts, but members of the professional categories too, and also invited people. At times of very serious occurrences or as part of campaigns, unions organize protest marches in places with great concentrations of people. The objective of such activity is not only class mobilization, but to air grievances in public, either directly or by means of the press coverage of such events.

#### 3.4. Education

Unions provide courses for members of internal accident prevention committees, with programs aimed at providing them with the capabilities for fighting for better working conditions and health. In one union, education begins with preparatory meetings involving all members of the internal accident prevention committees, followed by seminars that are divided into two degrees of complexity. In another union, courses take place annually and, in the year before the interview, these had brought together about 1,400 people.

The training material available for internal accident prevention committee activists is very extensive. The production of manuals and pamphlets that give guidance on actions and present legal and technical matters is very common.

#### 3.5. Strengthening of organizations within the workplace

A constant feature of workers’ organizations within the labor movement that was studied was the strengthening of these organizations within the workplace. In this respect, internal accident prevention committees merited a special mention within unions’ work. There were countless citations in interviews and the documents collected regarding the actions of internal accident prevention committees.

In a report edited by the Metal Workers’ Union of São Paulo,^32^ the issue is discussed with the professional category in the following transcript:

*"One of the instruments that workers have in their workplace for improving the environmental conditions at the plant where they work is the internal accident prevention committee. That is why our Union has invested in training as well as having discussions with workers about how important participation is and about the election of our fellow workers who are committed not only to the business but also to bringing in solutions for the many problems found in the working environment. If you are elected as a member of an internal accident prevention committee, you should contact the Union to get new instructions or to bring us information about accidents occurring in your factory."*

An important characteristic of union undertakings is that enterprises representing major interests, either due to employee numbers or political position in relation to the class setting, have more union activities organized, with directors especially chosen for these tasks. Part of their task is to observe and identify workers who are outstanding in their interest in and political commitment to health and safety at work, for when election time for the internal accident prevention committee comes. Following candidate registration, name publicity work for the candidate begins, supported by the Union.

Some unions produce advertising material for candidate use. In others, assemblies are held in front of the factories and the candidates supported by the union have their names announced by loudspeakers. Most of the time, candidates supported by unions win the elections.

In elections for internal accident prevention committees, it is common for employees who are not committed to workers’ aspirations to be registered as candidates and be elected. These may or may not be the bosses, but they act on internal accident prevention committees to defend the interests of the enterprise. The unions denounce them and form lists in opposition to these representatives, but on many occasions, pressure caused by the power of the enterprise does not allow such action for change to have success.

The rules for the election process for internal accident prevention committees are often disrespected by employers, with candidates being fired after their registration, or before registration, when the actions of a future candidate come to notice. This problem is constantly complained about by unions, but frequently it is only resolved by the labor courts, long after the election has taken place. Activists on internal accident prevention committees whose actions have stood out are natural candidates for representing other union matters. However, because such employees are guaranteed permanent employment during their mandates, they become victims of dismissal by their companies, merely for having undertaken such actions.

#### 3.6. Collective bargaining

Bargaining about issues relating to health and working conditions is a priority for all the unions that were studied. There have been various gains in benefits for professional categories or by enterprises. In this type of bargaining, changes in the working environment and organization come into being, while in negotiations by professional categories the gains have been in the field of rights and regulations.

One of the union directors interviewed described bargaining that took place with the state telecommunications company and resulted in the shortening of telephone operators’ working day from eight to six hours and the installation of noise suppression devices on the telephone switchboard. Another union director described negotiations that resulted in the creation of an occupational diseases study committee, made up by employers and employees, which had the function of developing projects to target reductions in the incidence of work-related diseases among the professional category.

All salary campaigns list workers’ health issues in their claims, and these are progressively taking on more importance in bargaining, as reported by the union directors interviewed. This is especially so during periods of economic stability, when there is not much fighting for salary readjustments. Such lists of claims include mention of the laws and standards that are essential for the professional category, such as the constitutional right to refuse to do dangerous work and the Ministry of Labor’s norms that regulate data processing work.

#### 3.7. Inter-institutional action on workers’ health

The unions studied maintain relations with other entities that act on workers’ health. As observed during the interviews and in the material collected, inter-institutional relations regarding union action are extensive, and one of the concerns in these is to draw up joint policies and actions. In São Paulo, there is the Inter-institutional Forum for Workers’ Health, in which the unions are the leading players, and this takes on the fight for rights regarding workers’ health issues, especially against the National Social Security Institute, the government agency that is responsible for social security compensations and sickness benefits.

Unions’ satisfaction regarding inter-institutional action varies from region to region and from union to union. One union director from Minas Gerais, when interviewed, described the hard attitude encountered when the union participated in an inter-institutional forum with public institutions:

*"I perceive that we have to act more strongly with these organizations, because they have a strong inclination towards prioritizing the employer’s side. They say what the employers are saying. That is, they can’t meet the claims because of globalization or competition …"*

A union director from São Paulo spoke about this action:

*"From the moment you take the decision to work at an inter-institutional level, you have to work with these people. For us, from the union, it has been a big lesson and is very empowering, too. We went as far as a lockout with the petrochemical industry in 1992 and we concluded that it is worth working with these people*…"

Another union director from São Paulo, despite his union’s policy of encouraging inter-institutional action, stated that the policy is not bringing the expected benefits in relation to workers’ health.

#### 3.8. Structures for action on workers’ health

The unions studied have physical and service structures for action on workers’ health of a variety of sizes. Some set aside rooms for attending to union members, with professionals specially hired for this action, such as physicians, psychologists, phonoaudiologists and engineers, with date processing, press advisory and other facilities. Some unions do not have such structures for economic reasons.

Medical care for workers is being transferred to the public health sector. Since the end of the 1980s, the public sector has been establishing workers’ health programs that provide such attention.

#### 3.9. Influences on actions

In interviews and in the material collected, evidence has been sought regarding foreign influences on Brazilian unions’ actions on workers’ health. It was seen that the Italian workers’ model and the instruments proposed by Prof. Asa Cristina Laurell have had a remarkable influence on the Brazilian union movement. The difficulties in applying these were considered in the interviews, in particular those resulting from guarantees of union action and work organization, which are very different in the two countries. The Italian union influence was also referred to in a bulletin from the Chemicals and Petrochemicals Union of Bahia^33^ and in an interview with a union director:

*"Força Sindical is a member of the International Confederation of Free Union Organizations and, through Força Sindical, our union participates in all of the international confederation’s activities concerning health issues, including the International Federation of Metal Industry Workers. In October, we had a meeting with the French Force Ouvrière. They were here and we had a one-week course with them. It was about health issues. A course with the American Federation of La- bor/Congress of Industrial Organizations took place in Washington. We have had courses in Bolivia with Union General de Trabajadores and Comisiones Obreras, together with the Spanish Health Ministry. Then, we had this contact too. In spite of the fact that we do not have any relationship with the Italians, our material is all based on Italian things, too: non-delegated, homogeneous group risk-maps. Our material is all along these lines, from the Italian project."*

The Italian influence on Brazilian unions’ actions on health issues had a large contribution towards the founding of the National Institute for Health at Work, which was a result from the collaboration between Central Única dos Trabalhadores and Confederazione Generali Italiana dei Lavoratori. Visits by Brazilian union advisers and directors to other countries have become more frequent, with the aim of getting to know different aspects of inter-national union action.

## CONCLUSIONS

Union action on workers’ health in Brazil is restricted to some unions, located mainly in the southern, southeastern and northeastern regions of the country. In spite of this, the unions that undertake actions on health issues are those that represent professional categories with large numbers of members and which have great economic and political importance. These unions are organized around the most developed areas of the country. Although such actions are restricted to only a few entities, these unions’ characteristics give them wider scope and national importance, thus amplifying their impact on workers’ health actions developed by governmental and industrial bodies, and also having a positive influence on health-care professionals.

The recent changes in the health and safety at work regulations, the recognition of professional diseases, the creation of workers’ health services and programs in the Unified Health System and the improvement in the professional actions of specialized services for safety and occupational medicine within industries, all basically result from union action.

Through analysis of the way in which union action has taken place, it can be demonstrated that there is commonality in the methods of union action in the field of workers’ health. One component that is common to all such actions is the search for technical and political strengthening of all workers and their general and local representation, by means of:

- Worker information, with the objective of creating critical awareness regarding the health/sickness-at-work process;
- Worker mobilization regarding aspects of work-health relationships;
- The education of union activists regarding health at work.

The technical and political strengthening of unions has a beneficial objective in collective bargaining between employers and workers, and inter-institutional action on behalf of workers’ rights guarantees and amplifies the improvement of health and working conditions.

The Italian worker’s model and its instruments, as discussed and published especially by Prof. Asa Cristina Laurell, has had a remarkable influence on Brazilian unions’ methods for action on workers’ health, and this model has been transformed into regulations such as the obligation to draw up risk maps in all workplaces. The collaboration between Central Única dos Trabalhadores and Confederazione Generali Italiana dei Lavoratori has reinforced this influence.
